# Adhesion and Interfacial Interactions Promoted by Tannic Acid and 1,2,3,4-Butanetetracarboxylic Acid in Casein/Carboxymethylcellulose Bilayer Films

**DOI:** 10.1021/acs.langmuir.5c01942

**Published:** 2025-07-14

**Authors:** Giuliana T. Franco, Luana Figueiredo, Caio G. Otoni, Luiz H. C. Mattoso

**Affiliations:** † Nanotechnology National Laboratory for Agriculture (LNNA), Embrapa Instrumentation, Rua XV de Novembro, 1452, São Carlos, São Paulo 13561-206, Brazil; ‡ Department of Chemistry, Federal University of São Carlos (UFSCar), Rod. Washington Luís, km 235, São Carlos, São Paulo 13565-905, Brazil; § Graduate Program in Chemistry (PPGQ), Federal University of São Carlos (UFSCar), Rod. Washington Luís, km 235, São Carlos, São Paulo 13565-905, Brazil; ∥ São Carlos Institute of Chemistry (IQSC), University of São Paulo (USP), Av. Trabalhador São Carlense, Parque Arnold Schimidt, São Carlos, São Paulo 13566-590, Brazil; ⊥ Graduate Program in Materials Science and Engineering (PPGCEM), Federal University of São Carlos, Rod. Washington Luís, km 235, São Carlos, São Paulo 13565-905, Brazil; # Institute of Chemistry, University of Campinas (Unicamp), Rua Monteiro Lobato, 270, Campinas, São Paulo 13083-862, Brazil

## Abstract

The design of functional multilayer materials relies on interfacial phenomena that govern component compatibility, structural integrity, and material performance. Self-supporting films were produced by combining casein and carboxymethylcellulose (CMC) bilayer. Here, we incorporated tannic acid (TA) and 1,2,3,4-butanetetracarboxylic acid (BTCA) into CMC layers as protein- and polysaccharide-crosslinking additives at varying ratios to tailor interfacial energy. The incorporation of TA led to interlayer adhesion, as evidenced by the increased force required for bilayer delamination. Additionally, the reduced moisture adsorption capacity upon TA addition supports the occurrence of interfacial crosslinking with the casein layer. ATR–FTIR measurements in the delaminated faces suggested a structural rearrangement that exposed nonpolar groups at the interfacial region. The vOCG approach predicted the contributions of secondary interactions, revealing that BTCA enhances electrostatic interactions, while TA contributes to nonpolar interactions. The theoretical work of adhesion was modulated according to the proportions of both crosslinkers. Understanding interfacial interactions helps design biobased materials with tailored properties and suitable performance as sustainable alternatives to plastics in the circular bioeconomy.

## Graphical abstract

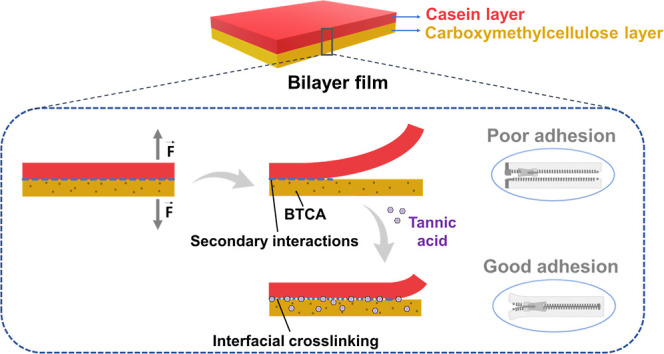

## Introduction

Natural polymers have been explored to develop materials aligned with the principles of the circular economy. However, the actual application of several biobased materials is restricted by technical constraints, often mechanical and barrier properties, or solvent resistance.^1^ Strategies to overcome such limitations include combining materials in the form of blends,^2,3^ coacervates,^4,5^ multistructured systems,^6,7^ and nanocomposites.^8,9^ If heterogeneous, interfaces denote a critical aspect to consider as they determine key properties of multiphase materials.^10^ In this sense, surface phenomena are to be controlled to achieve suitable thermodynamic compatibility, adhesion, adsorption, and interaction toward component synergism.^11,12^ Multistructured films are emerging as promising materials for various applications, including food packaging,^6^ stimuli-responsive films,^13^ and drug delivery systems,^14^ due to their ability to combine multiple functional properties within a single material.

Proteins and polysaccharides are widely used as raw materials due to their chemical diversity and versatility, which are usually integrated into other polymers.^15–17^ Carboxymethylcellulose (CMC) is a water-soluble anionic polysaccharide derived from cellulose with good mechanical properties.^18^ Casein is a phosphoprotein classified as an intrinsically disordered protein (IDP) due to the absence of secondary and tertiary structures, which allows its organization as a micelle. Due to its amphiphilic character, casein can change its conformational structure depending on the chemical environment and the surface on which it is adsorbed.^19,20^ Casein and CMC are commonly combined as complex coacervates, which exhibit high stability and a compact structure, allowing their application as carriers for active compounds such as anthocyanins and curcumin.^21–23^ Souza et al.^24^ demonstrated that physicochemical and morphological properties can be tailored by pH and casein/CMC proportions.

Cross-linkers are often employed to enhance functional properties and the compatibilization of polymeric materials.^25,26^ Among them, 1,2,3,4-butanetetracarboxylic acid (BTCA) is a poly­(carboxylic acid) commonly used for crosslinking mainly cellulose and its derivatives, improving its moisture resistance. The reaction mechanism is given by forming ester bonds between the carboxyl of the anhydride derived from BTCA and the hydroxyls of the polysaccharide.^17^ Polymer combinations may require incorporating compatibilizing agents to modify surface energy and promote interfacial interactions.^11^ Tannic acid (TA) is a phenolic compound that can bind the protein chains by multiple covalent bonds between tannin hydroxyls and protein amines.^17^ These bonds are formed mainly with lysine, tyrosine, and cysteine residues under alkaline conditions and in the presence of oxygen.^27^ TA has been used as a natural compatibilizer for proteins and polysaccharides, mainly in food applications. The literature shows that this polyphenol forms stable chemical crosslinks^28^ and provides additional properties, such as antimicrobial,^29^ antioxidant,^30^ and food preservation effects.^31^ Thus, TA was used to promote crosslinking at the CMC-casein interface film, improving its adhesion. The secondary interactions also contribute to surface phenomena, but those cannot be measured directly.^32^ For that, theoretical approaches are applied to study these effects.

The theoretical model proposed by van Oss, Chaudhury, and Good (vOCG) describes the molecular interactions at the solid–liquid interface based on the donation/acceptation of electrons, predicting nonpolar, polar, and acid–base interactions.^33^ The vOCG model expands the classical Lifshitz–van der Waals (LW) theory by incorporating acid–base interactions in addition to dispersive (nonpolar) interactions, thereby enabling the estimation of the contributions of secondary interactions (e.g., van der Waals, polar, and acid–base forces) to the surface energy.^34^

Insufficient interlayer adhesion represents a critical challenge in the bistructured materials, hindering their functional performance and the expression of the combined properties. In this work, the interfacial interactions between CMC and casein, integrated in a self-supporting bilayer film, were investigated. In addition to BTCA, TA was incorporated into the CMC layer to enhance the interfacial adhesion through interfacial crosslinking. The study further assessed the effects of TA and BTCA crosslinker proportions on the interlayer interactions. The contributions of nonpolar, polar, and acid–base forces were also studied using the vOCG theoretical model.

## Materials and Methods

### Materials

Carboxymethylcellulose sodium salt, CMC (USP; Synth, Brazil; degree of substitution: 0.95 ± 0.09); casein sodium salt, CA (80% pure; Exôdo Científica, Brazil); glycerol (99.5% pure; Dinâmica, Brazil); tannic acid, TA (ACS; Sigma-Aldrich, China), 1,2,3,4-butanetetracarboxylic acid, BTCA (99% pure; Sigma-Aldrich, India); sodium hypophosphite, SHP (99%; Synth, Brazil); and toluene (99.5% pure; LS Chemicals, Brazil) were used as received. Ultrapure water, deionized (ρ = 18.2 MΩ cm) in a Milli-Q system (Barnstead Nanopure Diamond, USA), was used in all experiments.

### Methodology

#### Film-Forming Solutions

The CMC layer was prepared by solubilizing the polymer in water at 2 wt %, wet basis (w.b.), and 60 °C. Then, BTCA and SHP were added at a 2:1 weight ratio, accounting for 0, 15, or 30 wt %, dry basis (d.b.), predissolved in 3 mL of water, and stirred for 10 min. TA was added at 0, 5, or 30 wt %, d.b., predissolved in 3 mL of water, under stirring for 30 min, followed by the addition of 15 wt % glycerol, d.b., under another 30 min of stirring. The solution was centrifuged at 7000 rpm for 10 min to remove air bubbles. The CMC films were prepared at the following wt % of BTCA/TA, based on dry CMC mass: 15/0, 15/5, 15/30, 30/30, and 0/30. Casein was solubilized in water at 20 wt %, w.b., and pH > 5, adjusted with 3 M NaOH. Glycerol at 30 wt %, d.b., was added, and the system was stirred for 30 min at room temperature. The pH was adjusted to 8 before the solution was centrifuged at 7000 rpm for 10 min.

#### Bilayer Film Production

Self-supporting CA-CMC bilayer films were produced by continuous casting on a KTF-S coater machine (Werner Mathis AG, Switzerland). First, the CMC solution was poured onto a polyester substrate (Mylar, DuPont, Brazil) moving at 0.07 m min^–1^ toward a doctor blade device with a gap (wet thickness) set at 1.5 mm and two sequential convective ovens (1 m each) at 95 °C for drying. Then, the casein solution was deposited on the dried CMC layer, which moved at 0.1 m min^–1^ toward the doctor blade set at 0.8 mm and two convective ovens at 70 °C. Casein and CMC monolayer films were made for comparison. Once dried, all samples were conditioned at 50% RH and 25 °C for at least 48 h before testing.

#### Water Contact Angle and Surface Free Energy

The sessile drop method was used to determine the surface free energy (SFE, γ_S_). The contact angle was measured using a Theta Lite tensiometer (Biolin Scientific, Sweden) with 300 μL drops of three probe liquids with known surface energies, namely water, glycerol, and toluene (see Table S1). The basic thermodynamic equation to determine SFE is based on the vOCG model (eqs 1–3).^35^

1 $$\gamma_{L}\left(1+\cos\theta \right)=2\sqrt{\gamma_{S}^{LW}\gamma_{L}^{LW}}+2\sqrt{\gamma_{S}^{+}\gamma_{L}^{-}}+2\sqrt{\gamma_{S}^{-}\gamma_{L}^{+}}$$

2 $$\gamma_{S}=\gamma_{S}^{LW}+\gamma_{S}^{AB}$$

3 $$\gamma_{S}^{AB}=2\sqrt{\gamma_{S}^{+}\gamma_{S}^{-}}$$

where θ is the experimental contact angle, γ is the total surface tension (mJ m^–1^), γ^LW^ is the Liftshitz-van der Waals surface tension component (mJ m^–1^) (nonpolar component), γ^AB^ is the Lewis acid–base surface tension component (mJ m^–1^) (polar component), and γ^+^ and γ^–^ are the acid and base Lewis components (mJ m^–1^), respectively. The subscripts S and L refer to solid and liquid phases.

The work of adhesion between two solids, casein and CMC layers, was estimated by eqs 4–6.^36,37^

4 $$W_{a}=W_{a}^{AB}+W_{a}^{LW}$$

5 $$W_{a}^{AB}=2\sqrt{\gamma_{1}^{+}\gamma_{2}^{-}}+2\sqrt{\gamma_{1}^{-}\gamma_{2}^{+}}$$

6 $$W_{a}^{LW}=2\sqrt{\gamma_{1}^{LW}\gamma_{2}^{LW}}$$

where *W*_a_ is the total work of adhesion (J m^–2^), *W*_a_^AB^ is the Lewis acid–base work of adhesion component (J m^–2^), and *W*_a_^LW^ is the Lifshitz–van der Waals work of adhesion (J m^–2^).

The water contact angles for both the casein and CMC faces of the bilayer films were measured using the same parameters described to compare their hydrophilic/hydrophobic characteristics.

#### Spectroscopical Analysis

Fourier-transform infrared spectroscopy (FTIR) spectra were acquired on a Vertex 70 device (Bruker, Germany) operating in the attenuated total reflectance (ATR) and transmittance modes. Wavenumbers from 400 to 4000 cm^–1^ were scanned with a spectral resolution of 4 cm^–1^, and 32 scans were accumulated per sample. The intensity of the 923 cm^–1^ band was used to normalize all spectra.

#### Field-Emission Scanning Electron Microscopy

The morphology of the bilayer films was observed by scanning electron microscopy on a JMS 6510 device (JEOL, Japan). For cross-sectional images, the films were cryo-fractured under liquid nitrogen, fixed at 90° onto aluminum stubs, and coated with a thin platinum layer.

#### Equilibrium Moisture Content

The moisture absorption capacity of the bilayer films was investigated by keeping the samples (2 × 2 cm^2^) in a humidity-controlled desiccator at 50% RH and 25 °C for 24 h. The equilibrium moisture content (EMC) was determined according to eq 7.^38^

7 $$EMC\;\left(\%\right)=\frac{M_{f}-M_{i}}{M_{i}}\times 100\%$$

where EMC is the equilibrium moisture content (%), *M*_i_ is the initial mass (g), and *M*_f_ is the final mass (g).

#### Water Vapor Permeability

The water vapor permeability (WVP) was determined based on a modification of ASTM E96-80.^39^ The films (2 × 2 cm^2^) were mounted onto a sealed PTFE cup with an internal diameter of 2.4 cm filled with 2 mL water. For both casein and CMC layers, the external faces were mounted facing the interior of the test cell filled with water (i.e., high-RH environment). The test sets were kept at 25 °C and 50% RH (Ethik, 420–2TS 295L, Brazil) and weighed six times every hour and once after 24 h. The WVP was calculated by eq 8.

8 $$WVP=\frac{WVTR\times L}{\Delta P}$$

where WVP is water vapor permeability (g m h^–1^ Pa^–1^), WVTR is water vapor transmission rate (g h^–1^), *L* is the average thickness (mm), and Δ*P* is the pressure difference (kPa).

#### Mechanical Delamination Test

The qualitative delamination test was conducted by applying adhesive tape (Scotch 9400-3 M) on each face and peeling the layers apart (Figure 1). The maximum force required to delaminate the layers was determined according to the ASTM F904-16 standard using a TA.XT plus texture analyzer (Extralab Brasil, Brazil). Five specimens (2.5 × 25 cm^2^) were manually separated into layers, and each ply was stuck in both clamps. The initial distance between the champs was 2.54 cm, and the test speed was 2.8 cm min^–1^.

**Figure 1:**
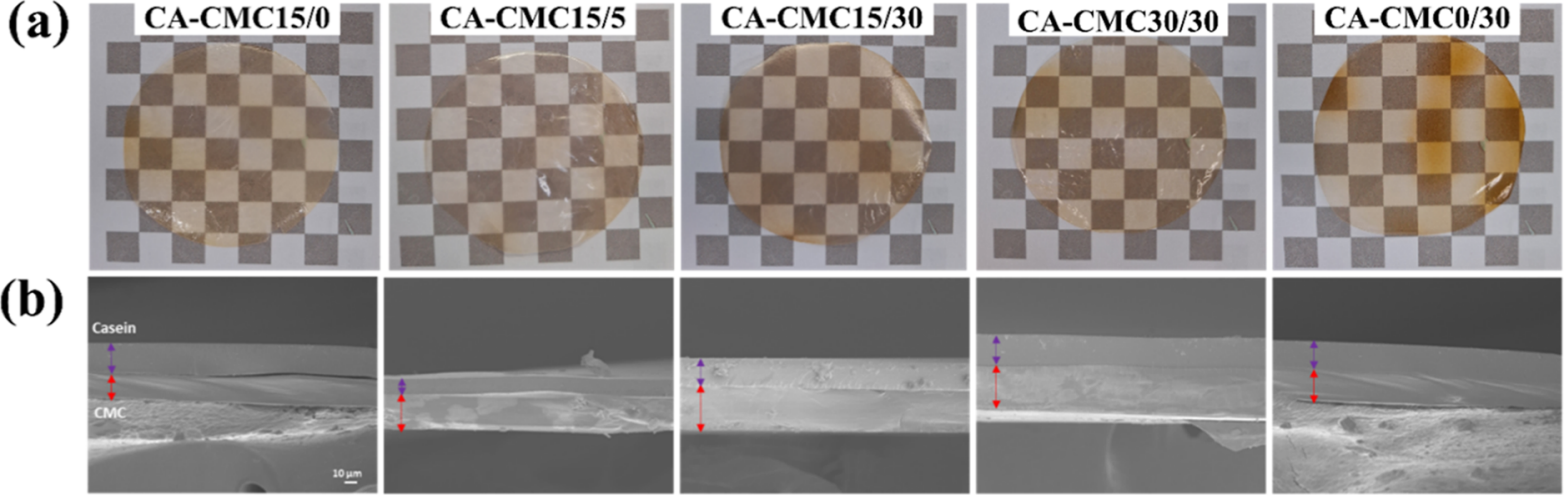
(a) Self-supporting bilayer films of casein and carboxymethylcellulose (CA-CMC) containing different proportions of 1,2,3,4-butanetetracarboxylic acid (*X*) and tannic acid (*Y*), *X*/*Y* in the sample label (background square edges = 246 × 185 mm^2^), and the respective (b) cross-sectional SEM images of the cryo-fractured bilayer films.

#### Statistical Analysis

The data were analyzed through analysis of variance (ANOVA) and Tukey’s test at 5% significance (*p* = *0*.*05*) using the Minitab software, version 18 (LLC, USA).

## Results and Discussion

### Adhesion in Casein-Carboxymethylcellulose Bilayer Films

The films were self-supporting, macroscopically continuous, optically transparent, and yellowish due to TA, as shown in Figure 1a. The BTCA was incorporated into the CMC layer to cross-link the polysaccharide layer and improve its moisture resistance since CMC is a highly water-soluble polymer.

SEM images of the cross-section surfaces (Figure 1b) show homogeneous morphologies. The differences in contrast allow distinguishing the protein and polysaccharide layers and delimiting the interface. No delamination was observed even at such a magnification, except for CA-CMC15/0, corroborating the macroscopic observations. Indeed, images of the external (noninterfacing) and delaminated (interfacing) faces indicate that the deposition of the second layer modifies the surface aspect of the substrate layer (see Figure S1). Crosslinking arises from esterification reactions between carboxyl groups of BTCA and hydroxyl groups of CMC.^40^ The bilayer films containing only BTCA as a crosslinker (CA-CMC15/0) were easily delaminated when handled, owing to the low interlayer adhesion (Figure 2a). TA was also added to the CMC layer, where it interacts primarily through secondary interactions (e.g., hydrogen bonding, electrostatic forces, and hydrophobic interactions, as indicated by the ^13^C NMR spectra^41^ (see Figure S2). Additionally, the polyphenol serves to anchor the casein layer and improve interlayer adhesion, as evidenced by the Raman spectra (see Figure S3). The TA can react with amine groups to form C–N bonds—mainly lysine, tyrosine, and cysteine residues—by Michael reaction or Schiff base addition.^17^ Physical anchoring can also contribute to adhesion. A schematic illustration of the interfacial interactions is shown in Figure 2b. Indeed, the layers could no longer be manually delaminated after TA incorporation (Figure 2c,d). Previous tests showed that incorporating TA in the casein layer does not promote the same effect.

**Figure 2:**
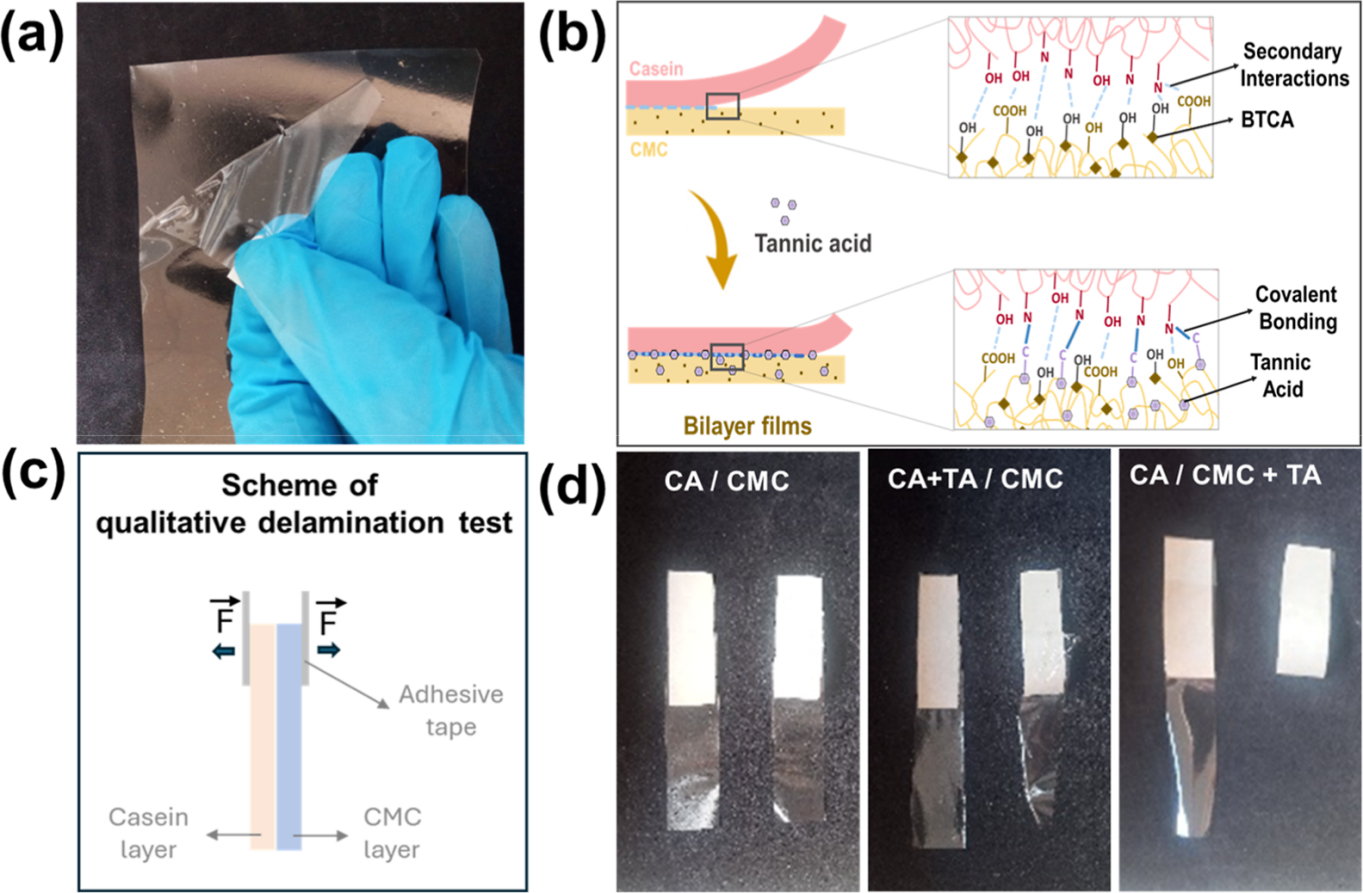
(a) Image of the manual delamination of bilayer films without incorporating tannic acid (CA-CMC15/0), (b) a schematic illustration of interfacial interactions promoted by 1,2,3,4-butanetetracarboxylic acid (BTCA) and tannic acid (TA) between casein and carboxymethylcellulose (CMC) layers, (c) scheme of the qualitative delamination test, and (d) images of bilayer films of CMC and casein without the addition of TA (CA/CMC), with the addition of TA in the casein layer (CA + TA/CMC), and with the addition of TA in the CMC layer (CA/CMC + TA) after the delamination.

To assess the influence and contribution of each crosslinker to the interfacial interactions, various proportions of BTCA and TA in the CMC layer were evaluated. The CA-CMC15/0 films showed an EMC of 38% (Figure 3a), which decreased to 10% with the incorporation of TA into the CMC layer. Slight changes in moisture uptake are observed according to the amount of TA and BTCA added. The mechanical delamination test provided the required force to separate the layers (Figure 3a), although with high deviations due to the nonuniformity of interfacial adherence. The CA-CMC15/5 film had an increase of 106% in the force required for delamination compared to the TA-free film (CA-CMC15/0), suggesting that polyphenol promoted interfacial interactions. However, increasing the concentration of BTCA and TA (CA-CMC15/30 and 30/30) seems to disfavor interlayer adherence.

**Figure 3:**
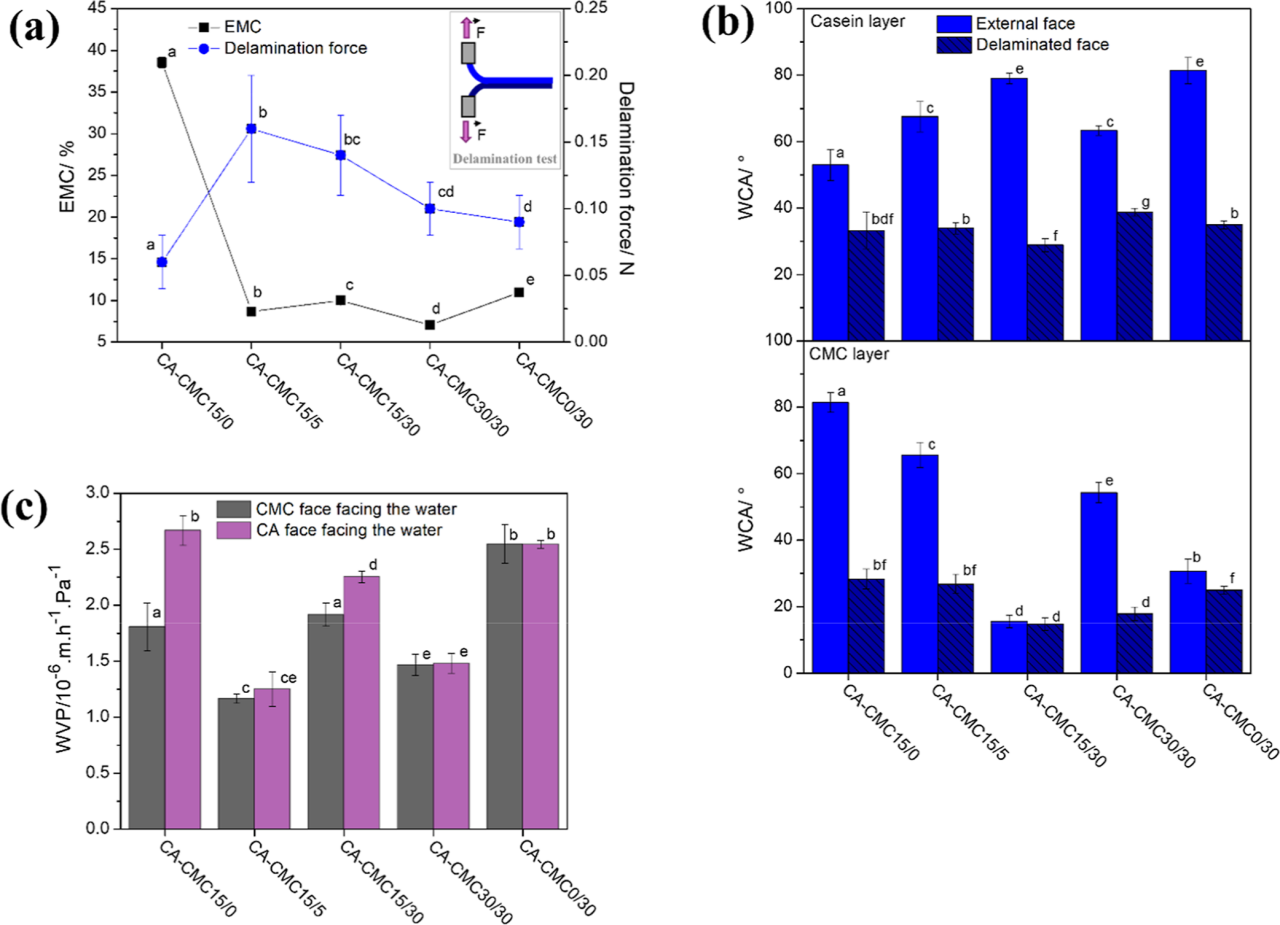
(a) Correlation between the equilibrium moisture content (EMC) and the delamination forces of bilayer films, (b) water contact angles (WCA) of external and delaminated faces of carboxymethylcellulose and casein layers, and (c) water vapor permeability (WVP) of bilayer films. Different letters indicate statistically significant differences (*p* < 0.05).

It is worth mentioning that the CA-CMC0/30 film, containing only TA as a crosslinker, demonstrated 30% higher adhesion than that containing only BTCA, supporting the effect of TA at the system interface. The opposite behaviors between the EMC and the delamination force highlight the role of TA in promoting anchoring points between casein and CMC layers, possibly through covalent bonds and polar interactions, which hinder moisture accommodation. This is supported if considering that the introduction of the hydroxyl-rich tannin would result in a greater affinity to water molecules^42^ and enhance moisture uptake, as indicated by the greater susceptibility to wettability in films containing TA (Figure 3b).

The hydrophilic/hydrophobic character changes were evaluated by water contact angle measurements of the external and delaminated faces of both layers (Figure 3b). Regardless of the material, the delaminated faces were significantly more hydrophilic (with lower contact angles with water) than the external faces. This behavior is attributed to the hypothesized preferential exposure of polar groups at the interface when the casein solution is deposited onto the dried CMC film.^19,43^ This effect is supported by the morphological changes between the external and delaminated faces (see Figure S1). The composition of crosslinkers affected the wettability of the CMC layers, in which the increase in BTCA concentration reduced the contact angle of the external faces from 65° (CA-CMC15/5) to 15° (CA-CMC15/30). Ostrowska-Czubenko et al.^44^ attributed this behavior to the reduced occurrence of hydrophilic groups capable of interacting with water, which were esterified with the crosslinker. Here, introducing TA also enhanced the wettability, as shown by the reduction of the contact angle according to the polyphenol addition and concentration. Furthermore, compared to BTCA, the studied polyphenol renders the film less wettable. Missio et al.^45^ observed the same effect with tannin-incorporated cellulose nanofiber films. The authors argued that tannin is a bulky molecule with hydrophilic and hydrophobic domains, and its hydrophilic sites interact with the hydrophilic sites of cellulose, resulting in the exposure of the hydrophobic sites, consequently reducing the surface affinity for water. However, the CA-CMC30/30 films showed that increasing the TA concentration stimulated the hydrophilic character of the external surface. This effect can be related to the high concentrations of BTCA and TA, which, after saturation of the hydrophilic sites in the matrix, leave polar groups available to interact with water. The phenomenon is frequently observed in films incorporated with nanocellulose, in which the nanostructures reduce the hydrophilic character until reaching a specific saturation concentration.^46^ The wettability of casein faces exhibited a low influence on bilayer composition because the same formulation was used for all systems.

The WVP of the films was evaluated when the external surfaces were exposed to the high-RH environment (Figure 3c). It was hypothesized that the layer (i.e., whether casein or CMC) facing the moisture-saturated environment could influence the permeability, given the different water affinities indicated by their contact angles. However, the results showed minimal impact from the surface, suggesting that moisture adsorption does not primarily drive the permeation process for these systems. Instead, the crosslinker proportion modulated water diffusion. Introducing TA reduced the film permeability to water vapor (vide CA-CMC15/5). Contrastingly, despite its capacity of crosslinking polysaccharides, increased BTCA content led to greater film permeabilities, which were balanced by increased TA concentration (vide CA-CMC30/30). Indeed, the lowest permeability was enabled by both crosslinkers, suggesting a synergistic effect. These behaviors corroborate the results observed in the EMC analysis (Figure 3a).

ATR–FTIR provided insights on the preferential exposure of functional groups and interfacial interactions (Figure 4). The typical bands observed for CMC layers are attributed to the stretching of hydroxyl groups and hydrogen bonding (−OH, ν = 3300 cm^–1^), asymmetric stretching of aliphatic methyl groups (−C–H, ν = 2900 cm^–1^), deformation of carboxylic acid groups (−COOH, ν = 1710 cm^–1^), stretching of carboxylate ions (−COO^–^, ν = 1595 cm^–1^ and 1410 cm^–1^), and bending of methyl groups (−CH, ν = 1318 cm^–1^).

**Figure 4:**
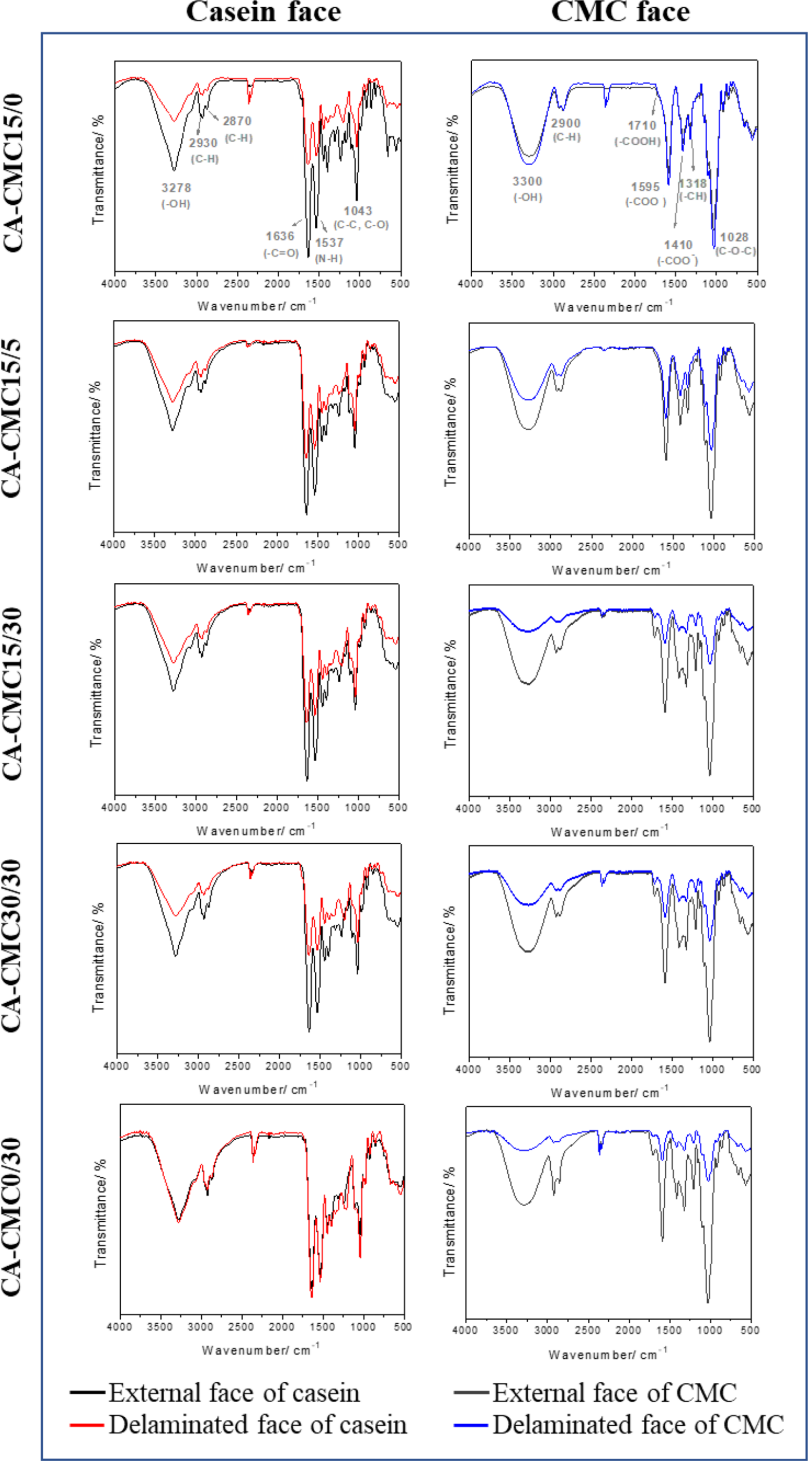
ATR–FTIR spectra of the external and delaminated faces of casein and carboxymethylcellulose (CMC) layers.

The cyclic backbone of the polysaccharide structure shows bands near 1105 cm^–1^, associated with C–O–C stretching vibrations, and around 1026 cm^–1^, related to ether bond stretching. Bands in the region between 1000 and 500 cm^–1^ are attributed to stretching and bending vibrations of −CH groups from the polysaccharide chains.^47–49^ Regarding casein, the relevant bands correspond to the stretching of hydroxyl groups (−OH, ν = 3278 cm^–1^), stretching of amide bonds (N–H, ν = 3285 cm^–1^), symmetric and asymmetric stretching of aliphatic methyl groups (−C–H, ν = 2930 and 2870 cm^–1^, respectively), amide I stretching (−C=O, ν = 1636 cm^–1^), amide II stretching (−N–H, ν = 1537 cm^–1^), and stretching of C–C and C–O bonds (1043 cm^–1^).^49–51^ Table 1 shows the variation of some characteristic band intensities (Δ*I = I*_delaminated face_ – *I*_external face_) between the external and delaminated faces to verify conformational changes. For the CMC layers, the examined bands were 3300 cm^–1^ (−OH), 1740 cm^–1^ (−COOH), and 1587 cm^–1^ (−COO^–^), while for the casein layers, the bands were 3278 cm^–1^ (−OH), 1636 cm^–1^ (−C–O), and 1537 cm^–1^ (−NH).

**Table 1:** Values of the differences in the intensities of typical ATR–FTIR bands for the external and delaminated faces (Δ*I* = I_delaminated face_ – I_external face_) of the carboxymethylcellulose (CMC) and casein layers.^a^ ^b^

	Δ*I* CMC faces/%			Δ*I* casein faces/%		
Bilayer films	3300 cm^–1^	1710 cm^–1^	1595 cm^–1^	3278 cm^–1^	1636 cm^–1^	1537 cm^–1^
CA-CMC15/0	30	0	40	–22	–7	21
CA-CMC15/5	43	–1	49	–49	–106	–74
CA-CMC15/30	–32	–6	–13	–44	–97	–58
CA-CMC30/30	–38	3	–61	10	–18	26
CA-CMC0/30	–32	–19	–43	42	77	70

a All intensities were normalized by the intensity of the invariant band at 923 cm^–1^.

b I: band intensities; *I* > 0 indicates a higher band intensity on the delaminated face than the external face; *I* < 0 indicates a lower band intensity on the delaminated face than the external face.

The comparison of spectra shows changes in band intensities among the external and delaminated faces of the CMC and casein layers (Figure 4), supporting the hypothesis of structural reorganization at the interface. The negative Δ*I* values for TA-incorporated CMC layers suggest a low polar group exposition on the delaminated face, which may be caused by internalization of these groups or breakage of bonds/interactions due to delamination. The CA-CMC15/0 film exhibited an opposite tendency, showing that the presence of TA affected the interfacial region. Jia et al.^52^ found that adding TA to the casein matrix significantly influences its structural conformation, which supports our observations.

There was a decrease in the Δ*I* of the characteristic bands for the casein layers, except for the CA-CMC0/30 film. In addition to the possibility of interfacial reorganization, shifts in the band positions referring to the stretching of −C–NH_2_ bonds (1315 cm^–1^) to shorter wavelengths (Figure 4) were observed, indicating the participation of these groups in interfacial interactions. These results emphasize the nonpolar groups/bonds contributions in the interlayer adhesion. Casein is a protein rich in valine, leucine, and phenylalanine residues with nonpolar side chains, which justifies such interactions.^53^ Those observations can be corroborated by the work of Chen et al.,^19^ who carried out a computer simulation to demonstrate the interactions at the interface of a starch and zein bilayer film. The authors showed changes in functional group conformation, orientation, and exposure according to the biopolymer interactions. However, the ATR–FTIR results contradict the discussion about contact angles on exposure to polar groups. However, it is necessary to consider that the contact angle measures the wettability capacity of a surface; this one, when in contact with water, can cause the mobility of polymer chains and exposure of hydrophilic groups.

### Estimation of Polar, Nonpolar, and Acid–Base Interactions by vOCG

The vOCG approach allows estimating the total surface free energy (SFE or γ_S_) based on two components, one associated with nonpolar interactions (γ_S_^LW^) and the other with polar interactions (γ_S_^AB^). γ_S_^AB^ can also be divided into electron-donor (γ_S_^−^) and electron-acceptor (γ_S_^+^) components.^54^ For low-energy solids, such as most polymers, SFE can be determined by measuring the contact angles (see Figure S4) of three solvents with distinct polarities and analyzing the results using the vOCG model. The films generally exhibited a contact angle trend of θ_glycerol_ < θ_water_ < θ_toluene_ (Figure 5), indicating a higher affinity of the films with the more polar solvents.

**Figure 5:**
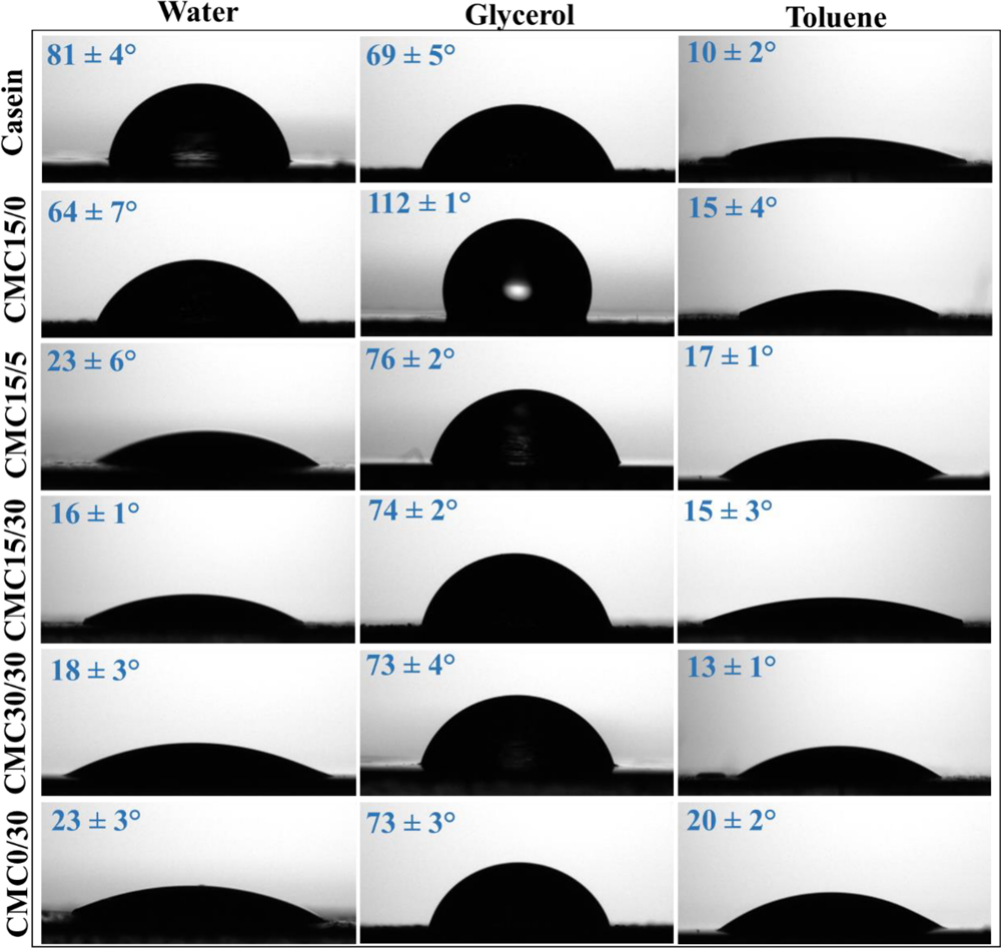
Contact angle values for different probe solvents. The equilibrium contact angles were considered at 30 s of analysis.

The results show that all films, except CMC15/0, have low surface energy (γ_S_ < 100 mN·m^–1^)^55^ (see Table S2). For CMC layers, the increase of the BTCA content tends to increase γ_S_, while the increase of the TA content decreases the surface energy. Thus, the γ_S_values result from combining the effects of both crosslinkers, and their amounts added. The CMC15/0 and CMC30/30 presented the highest and lowest γ_S_, respectively, while the other crosslinker proportions had subtle surface energy variations.

Figure 6 shows the correlation between γ_S_^LW^ and γ_S_^AB^, γ_S_^AB^/γ_S_^LW^ and γ_S_^AB^ and γ_S_^–^ and γ_S_^+^ components. CA films are governed by nonpolar interactions (Figure 6a), e.g., van der Waals and π–π stacks, which are related to the conformation of protein chains and exposed side chains.^56^ The CMC15/0 film shows a substantial contribution from polar interactions, e.g., hydrogen bonding, electrostatic, and dipole–dipole, which may be favored by the presence of polar groups and the formation of polar covalent bonds caused by the crosslinking of CMC and BTCA.^57^ For the other films, the contribution of both components was more balanced and did not present a behavior profile. Despite the apparent disconnection between the two components, the curve of γ_S_^AB^/γ_S_^LW^ as a function of γ_S_^AB^ (Figure 6b) shows a linear dependence relation. Equation 9 describes such behavior and allows to determine γ_S_^AB^ and γ_S_^LW^ values were used by measuring the contact angle for only one solvent.

9 $$\gamma_{S}^{AB}/\gamma_{S}^{LW}=0.019\pm 0.002\;\gamma_{S}^{AB}+0.4\pm 0.1$$

**Figure 6:**
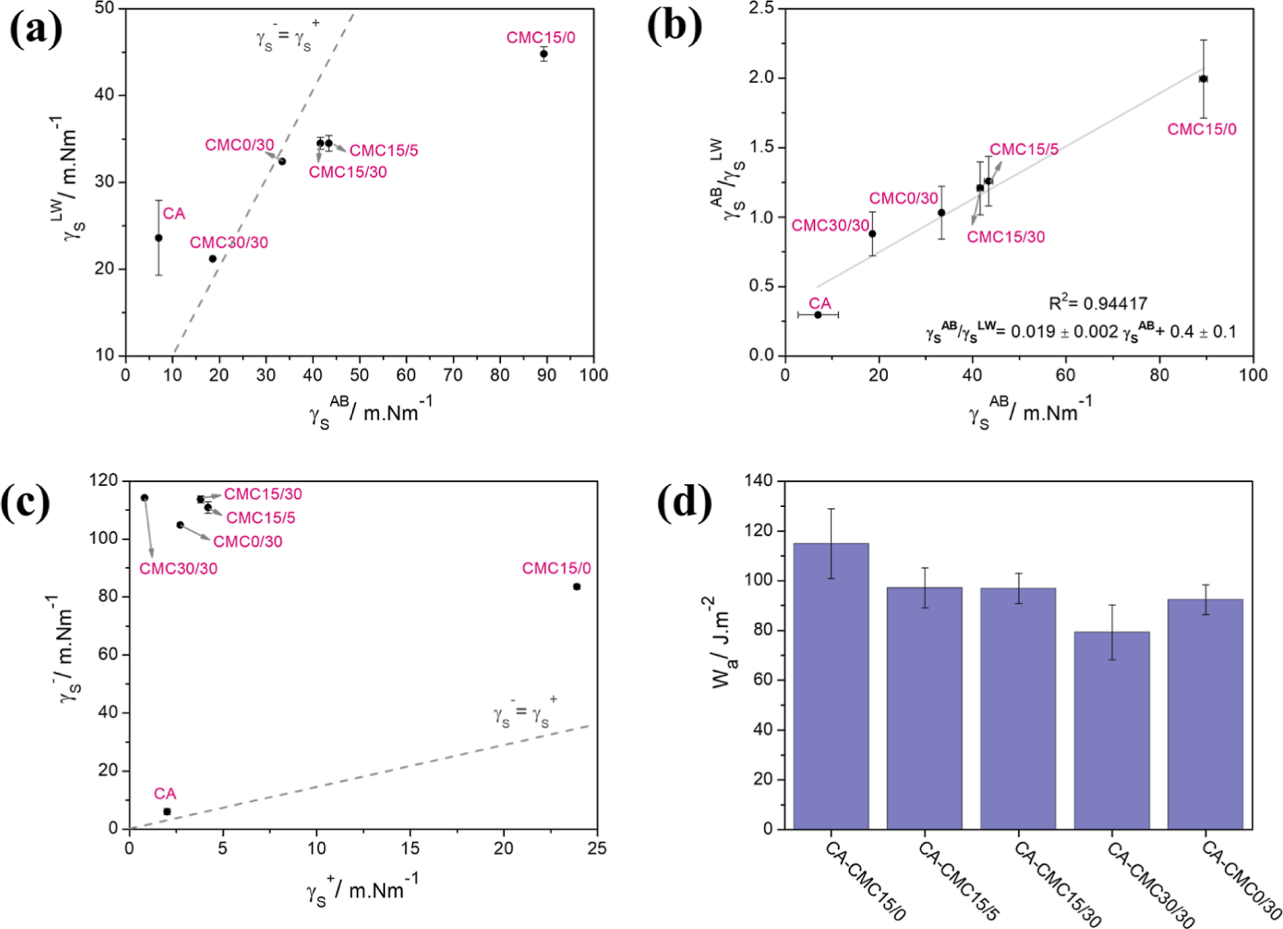
(a) Liftshitz-van der Waals component (γ_S_^LW^) plotted as a function of the Lewis acid–base component (γ_S_^AB^), (b) ratio of γ_S_^AB^/γ_S_^LW^ plotted as a function of γ_S_^AB^, (c) Lewis acid component (γ_S_^–^) plotted versus Lewis base component (γ_S_^+^), and (d) calculated values of total work of adhesion (*W*_a_) of bilayer films. The dotted line represents the curve γ_S_^AB^ = γ_S_^LW^ or γ_S_^–^ = γ_S_^+^.

The relation between γ_S_^+^ and γ_S_^–^ (Figure 6c) provides information about the acidity and basicity of surfaces and their ability to interact electrostatically. All CMC films exhibited a pronounced basic character (high γ_S_^–^) due to the high availability of carboxyl groups that can be deprotonated.^58^ The BTCA increases the electron-acceptor capacity, as observed by increasing the γ_S_^+^ value of CMC15/0 film, while TA induces the electron-receptor effect. The CA film showed a character close to neutrality, which may be related to the presence of amine and carboxyl groups that can be protonated/deprotonated as a function of the pH of the medium.^59^ Differences in the values, in magnitude, between acid–base components of casein and CMC films allow electrostatic interactions at the interface, which assist the adhesion between the biopolymer layers. It is important to note that this approach accounts only for the contributions of secondary interactions to surface energy, disregarding the formation of covalent bonds, which are hypothesized to play a key role in the interfacial adhesion between the layers.

The total work of adhesion (*W*_a_) and its components *W*_a_^LW^ and *W*_a_^AB^ (see Table S3) between the layers describe the surface’s ability to interact based on electrostatic, apolar, and hydrogen bonding interactions.^36^ The *W*_a_ calculated values (Figure 6d) showed that the interaction between CA and CMC is governed by nonpolar interactions (*W*_a_^LW^ > *W*_a_^AB^) which can be explained by the low exposure of polar groups at the casein layer interface, as observed in ATR-FTIR analysis. The CA-CMC15/0 film showed the highest *W*_a_, indicating that the interlayer adhesion is driven by electrostatic forces since CMC15/0 had the greatest electrostatic contribution in the surface energy. According to the model, the presence of TA disfavored the work of adhesion, while the BTCA stimulated the interaction between the layers. However, the CA-CMC15/0 bilayer is easily delaminated during handling (Figure 2a), while films containing TA incorporated into the CMC layer require more effort for delamination. It is important to note that the vOCG approach considers only the contributions of secondary interactions to surface energy calculation, disregarding the formation of covalent bonds, which are hypothesized to play a key role in the interfacial adhesion between the layers.

## Conclusions

Bilayer films were produced by combining casein and CMC through a continuous 2D interface. The incorporation of BTCA and TA into the CMC layer significantly affected the interlayer adhesion. While systems containing only BTCA exhibited low adhesion, the addition of 5% TA increased the delamination force by 106% and reduced moisture absorption by 74%, indicating strengthened interfacial interactions. ATR–FTIR spectroscopy revealed differences in band intensities between external and delaminated faces of both casein and CMC layers, indicating molecular reorganization at the interface. The proportions of BTCA and TA influenced the interchain cohesion and interfacial characteristics, modulating their water wettability and moisture permeation. The vOCG model predicted the contribution of secondary interactions of the work of adhesion between the layers. The approach showed that secondary interactions are more pronounced in the TA-free bilayers, although these systems exhibited weak interfacial adhesion. This study provides insights into how these crosslinkers modulate interfacial affinity and energy. Understanding interfacial interactions and their effects offers a toolbox for designing biobased materials with tailored properties, enhancing their commercial competitiveness and application potential.

## Supplementary Material



## Notes

SEM images of film’s faces; solid-state ^13^C NMR spectra; Raman spectra of casein layers, representation of the components of surface tension; and calculated values for surface free energy and work of adhesion (PDF)
