# Supplementary material for: Adhesion and Interfacial Interactions Promoted by Tannic Acid and 1,2,3,4-Butanetetracarboxylic Acid in Casein/Carboxymethylcellulose Bilayer Films
Source: Langmuir. 2025 Jul 14;41(29):19409–18. doi: 10.1021/acs.langmuir.5c01942 (PMC12312150; doi:10.1021/acs.langmuir.5c01942)
Supplement: Supplementary file 1 [file la5c01942_si_001.pdf]

Supporting Information

Adhesion and interfacial interactions promoted by tannic acid and 1,2,3,4-butanetetracarboxylic acid in casein/carboxymethylcellulose bilayer films

Author names:

Giuliana T. Franco[a,b,c*], Luana Figueiredo[a,d], Caio G. Otoni[e,f], Luiz H. C. Mattoso[a,c*]

Author affiliations:

[a] Nanotechnology National Laboratory for Agriculture (LNNA), Embrapa Instrumentation – Rua XV de Novembro, 1452, São Carlos, SP 13561-206, Brazil;

[b] Department of Chemistry, Federal University of São Carlos (UFSCar) – Rod. Washington Luís, km 235, São Carlos, SP 13565-905, Brazil;

[c] Graduate Program in Chemistry (PPGQ), Federal University of São Carlos (UFSCar) – Rod. Washington Luís, km 235, São Carlos, SP 13565-905, Brazil;

[d] São Carlos Institute of Chemistry (IQSC), University of São Paulo (USP) – Av. Trabalhador São Carlense, Parque Arnold Schimidt, São Carlos, SP 13566-590, Brazil.

[e] Graduate Program in Materials Science and Engineering (PPGCEM), Federal University of São Carlos – Rod. Washington Luís, km 235, São Carlos, SP 13565-905, Brazil;

[f] Institute of Chemistry, University of Campinas (Unicamp) – Rua Monteiro Lobato, 270, Campinas, SP 13083-862, Brazil.

Corresponding authors:

* Dr. Luiz Mattoso (luiz.mattoso@embrapa.br) and Dr. Giuliana T. Franco (gtfranco@estudante.ufscar.br).

List of content: Values of surface free energy for probe solvents; SEM images of external and delaminated faces of bilayer films; Solid-state ^{13}C NMR spectra; Raman Spectra; representation of three components of surface tension; calculated values of surface free energy; and calculated values of work of adhesion.

Table S1. Values of surface free energy (SFE, γ_S), Liftshitz-van der Waals component (γ_S^{LW}), and acid (γ_S^+) and base (γ_S^-) Lewis component (γ_S^{AB}), in $mN.m^{-1}$, of probe liquids.

	γ_L	γ_L^{LW}	γ_L^+	γ_L^-
Water	72.8	21.8	25.5	25.5
Glycerol	64	34	3.92	57.4
Toluene	28.3	28.3	0	2.7

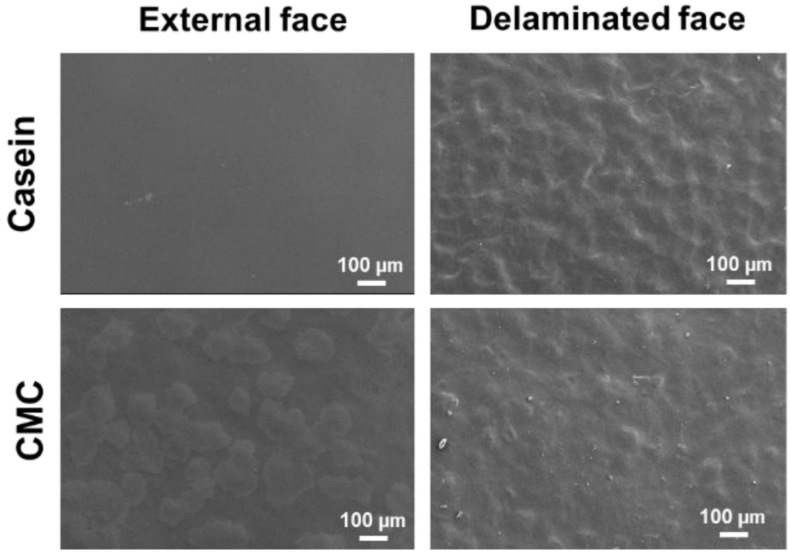

Figure S1. SEM images of the external (non-interfacing) and delaminated (interfacing) faces of casein (CA) and carboxymethylcellulose (CMC) layers after delamination of the film CA-CMC15/30.

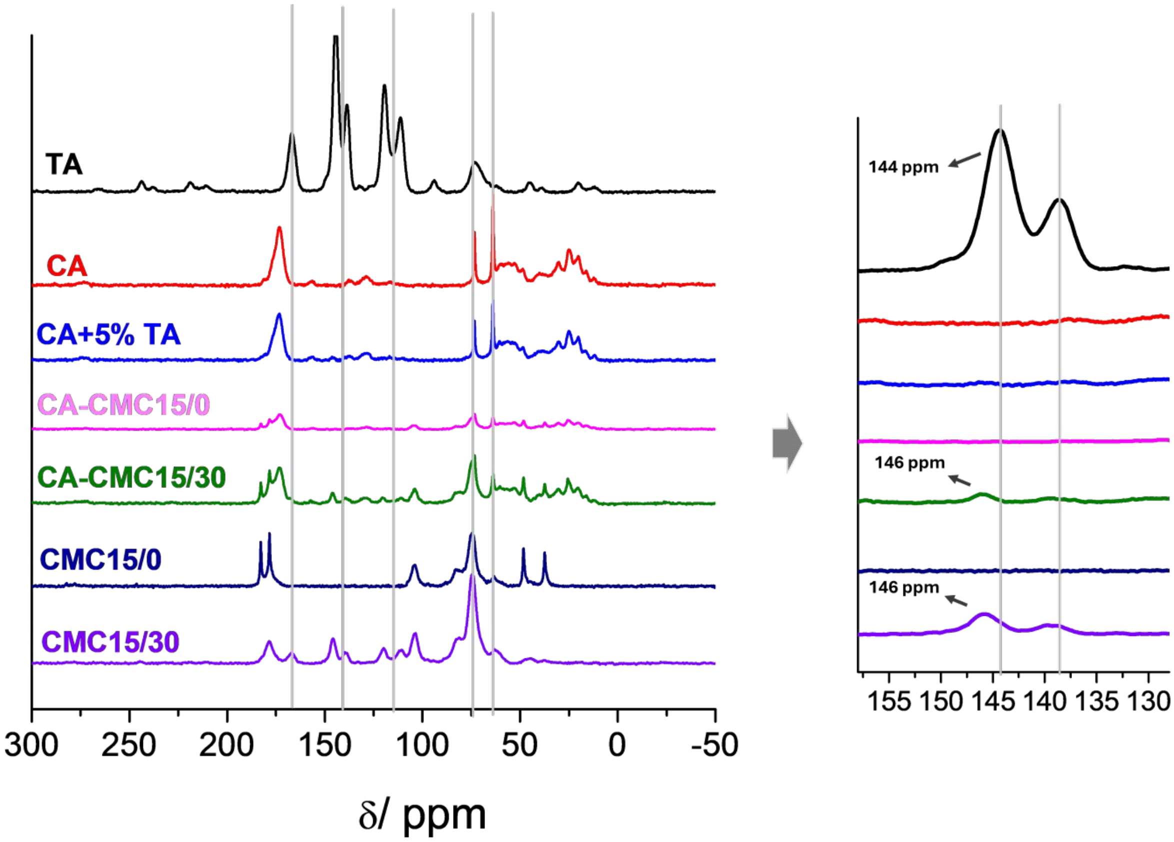

Figure S2. Solid-state ^{13}C NMR spectra for the following samples: tannic acid (TA) powder, casein (CA) monolayer, CA+5% TA monolayer, CA-CMC15/0 bilayer, CA-CMC15/30–bilayer, CMC15/0 monolayer, and CMC15/30 monolayer.

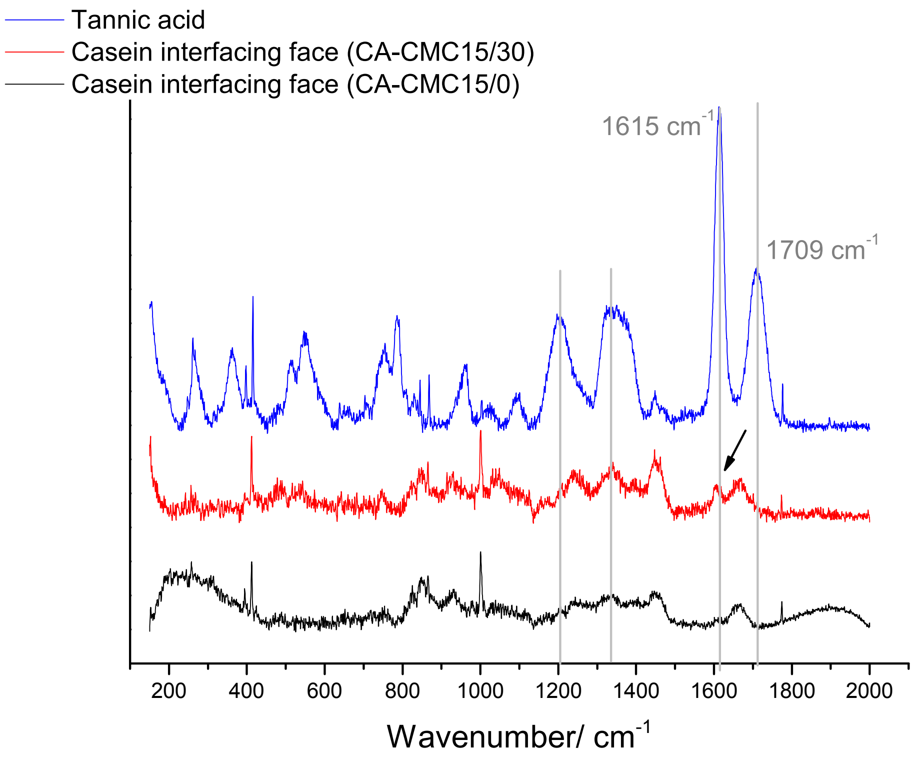

Figure S3. Raman spectra of the tannic acid and the delaminated casein face (interfacing face) of the bilayer films CA-CMC15/0 and CA-CMC15/30. The spectra were acquired using a 785 nm excitation wavelength.

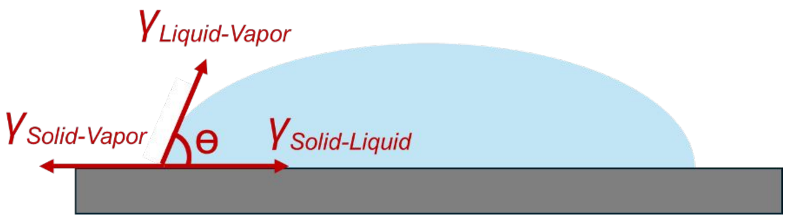

Figure S4. Schematic representation of the three components of surface tension (γ, surface energy) at the interface between a solid and liquid water.

Table S2. Calculated values for surface free energy (γ_{SV}), Liftshitz-van der Waals component (γ_{SV}^{LW}), and acid-base Lewis component (γ_{SV}^{AB}), in mN m^{-1}.

	γ_{SV}^{LW}	γ_{SV}^{AB}	γ_{SV}^{+}	γ_{SV}^{-}	γ_{SV}
Casein	23.6	7.0	2.0	6.0	30.6
CMC15/0	44.8	89.3	23.9	83.5	134.2
CMC15/5	34.5	43.4	4.2	110.9	77.8
CMC15/30	34.5	41.6	3.8	113.8	76.1
CMC30/30	21.2	18.6	0.8	114.3	39.8
CMC0/30	32.4	33.4	2.7	104.9	65.8

Table S3. Calculated values for the components of work of adhesion of polymeric films. W_a is the total work of adhesion; W_a^{AB} is the Lewis acid-base work of adhesion (polar component); W_a^{LW} is the Lifshitz-van der Waals work of adhesion (nonpolar component).

	W_a^{AB}	W_a^{LW}	W_a
CA-CMC15/0	49.9	65.0	114.9
CA-CMC15/5	40.0	57.1	97.1
CA-CMC15/30	39.9	57.1	96.9
CA-CMC30/30	34.6	44.7	79.3
CA-CMC0/30	37.1	55.3	92.4
